# Low‐Power Memristive Logic Device Enabled by Controllable Oxidation of 2D HfSe_2_ for In‐Memory Computing

**DOI:** 10.1002/advs.202005038

**Published:** 2021-05-29

**Authors:** Long Liu, Yi Li, Xiaodi Huang, Jia Chen, Zhe Yang, Kan‐Hao Xue, Ming Xu, Huawei Chen, Peng Zhou, Xiangshui Miao

**Affiliations:** ^1^ Wuhan National Laboratory for Optoelectronics School of Optical and Electronic Information Huazhong University of Science and Technology Wuhan 430074 China; ^2^ State Key Laboratory of ASIC and System School of Microelectronics Fudan University Shanghai 200433 China

**Keywords:** 2D HfSe_2_, in‐memory computing, low‐power consumption, memristors, oxidation, resistive switching

## Abstract

Memristive logic device is a promising unit for beyond von Neumann computing systems and 2D materials are widely used because of their controllable interfacial properties. Most of these 2D memristive devices, however, are made from semiconducting chalcogenides which fail to gate the off‐state current. To this end, a crossbar device using 2D HfSe_2_ is fabricated, and then the top layers are oxidized into “high‐*k*” dielectric HfSe*_x_* O*_y_* via oxygen plasma treatment, so that the cell resistance can be remarkably increased. This two‐terminal Ti/HfSe*_x_* O*_y_*/HfSe_2_/Au device exhibits excellent forming‐free resistive switching performance with high switching speed (<50 ns), low operation voltage (<3 V), large switching window (10^3^), and good data retention. Most importantly, the operation current and the power consumption reach 100 pA and 0.1 fJ to 0.1 pJ, much lower than other Hf—O based memristors. A functionally complete low‐power Boolean logic is experimentally demonstrated using the memristive device, allowing it in the application of energy‐efficient in‐memory computing.

## Graphical abstract

An energy‐efficient memristive device based on 2D HfSe_2_ oxides is fabricated, which is able to implement functionally complete Boolean logic with operation current down to 100 pA. The low‐power switching is realized by the formation and rupture of cone‐shaped O‐vacancy filaments in the ultrathin Hf−Se−O layer.

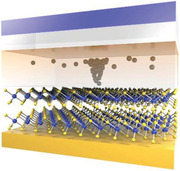

## 1 Introduction

Silicon logic transistors have served as the building blocks of the processing unit and memories in a traditional von Neumann system for over half a century. However, the pace of further improvement has been slowed down due to the discontinuation of the Moore's law. Meanwhile, as information technology embraces big data and artificial intelligence (AI), the von Neumann architecture inevitably encounters data transfer bottlenecks due to complex hierarchical structures. In recent years, researchers have been eagerly seeking for new materials and devices that could possibly replace or complement the traditional logic transistors to shift the von Neumann computing paradigm. Among many of them, the two‐terminal memristive device is considered as one of the most promising candidates for next‐generation high‐density nonvolatile memory and energy‐efficient in‐memory computing,^[^ ^1^, ^2^, ^3^, ^4^, ^5^, ^6^, ^7^, ^8^ ^]^ due to its high speed, low‐power consumption, high endurance, and the capability to collocate the memory and computing functions.^[^ ^9^, ^10^, ^11^ ^]^ Although remarkable progresses have been made in improving the performance of memristors, the most common transition metal oxides (TMOs, such as HfO*_x_* and TaO*_x_*) based devices still fail to meet the demand for energy‐efficient memory and computation tasks. To date, the operation current of such memristive devices remains at 10–100 µA level.^[^ ^12^, ^13^, ^14^ ^]^ Further decreasing the operation current to sub‐µA or even less is essential to reduce the energy consumption, particularly in some applications such as edge computing that requires to be extremely power efficient.^[^ ^15^ ^]^

To solve the above issues posed by bulk oxides, the 2D materials with controllable van der Waals (vdW) gaps and interfaces have recently shown their great potential in memristors due to their excellent mechanical and electrical properties.^[^ ^16^, ^17^, ^18^, ^19^ ^]^ The 2D layered materials are naturally good resistive switching (RS) media, particularly in the applications of energy‐efficient memory and computing due to its low physical dimensions. For example, Yan et al.^[^ ^20^ ^]^ reported a Pd/WS_2_/Pt device with an operation current of 1 µA, and Wang et al.^[^ ^21^ ^]^ further reduced the current down to 100 nA in the 2H‐MoS_2_ nanosheet memristor. However, most of such devices were made from chalcogenides which are less compatible with the semiconductor processing line than the oxides.^[^ ^22^, ^23^ ^]^ To this end, we devised a 2D‐like memristive oxide, starting from the transition metal dichalcogenide HfSe_2_, in which thin layers could be obtained easily through mechanical exfoliation.^[^ ^24^ ^]^ Unfortunately, this semiconducting chalcogenide cannot be directly used as the low‐power RS medium due to its relatively high conductivity,^[^ ^25^, ^26^ ^]^ failing to generate large resistance window upon switching. We notice that HfSe_2_, like black phosphorus, is not a very stable material, and even in air environment, the top layers of HfSe_2_ could be oxidized into “high‐*k*” dielectrics HfO*_x_* spontaneously.^[^ ^27^, ^28^ ^]^ To improve the quality of the oxide layer, we intentionally adopted the oxygen plasma (O_2_ plasma) treatment to control the oxidation time and layer thickness. The oxide layer fabricated using this method could induce high resistance and low operation current.

We then fabricated a vertical memristive device based on the above HfSe_2_ oxides. By using O_2_‐plasma treatment, a few top layers of HfSe_2_ are transformed into HfSe*_x_* O*_y_*, which serves as the RS medium of memristors. This Ti/HfSe*_x_* O*_y_*/HfSe_2_/Au device exhibits excellent forming‐free RS behavior with low voltage (<3 V), large switching window (>10^3^), and good retention characteristics (15 000 s). More importantly, this device shows ultralow operation current (100 pA) and power consumption (0.1 fJ to 0.1 pJ). The mechanism of the RS behavior is proposed and supported by the transmission electron microscopy (TEM). Our device is able to implement functionally complete low‐power Boolean logic, providing a feasible bottom‐up strategy to design novel low‐dimensional logic devices that operate in the energy level of sub‐pJ.

## 2 Results and Discussion

### 2.1 Design and Fabrication of HfSe_2_ Oxide Memristor

**Figure 1:**
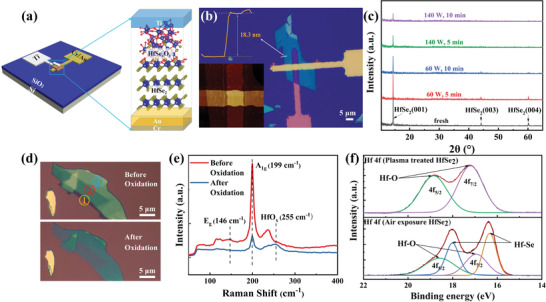
Fabrication and characterizations of HfSe_2_ oxide films and devices. a) Schematic illustration of Ti/HfSe*_x_* O*_y_*/HfSe_2_/Au memristor. b) Optical image of the fabricated device. The crosspoint area is 5 × 5 µm^2^. The inset images show AFM scan of the crosspoint (left lower panel) and AFM height profile (left upper panel) across the white line. c) XRD patterns of the HfSe_2_ nanosheets on the SiO_2_ substrate. d) Optical microscopy images of an exfoliated HfSe_2_ flake with different thicknesses. The transparency of the flake increases after O_2_‐plasma treatment, indicating the formation of Hf—O layer. e) Room‐temperature Raman spectra before and after 5 min O_2_‐plasma treatment of HfSe_2_ using a 532 nm laser. f) XPS spectra of Hf 4f core level for air‐exposed and O_2_‐plasma‐treated HfSe_2_ flakes.

Our memristive devices were built in a vertical crossbar form, as shown schematically in **Figure** **1a**. A very thin HfSe*_x_* O*_y_* (0 ≤ *x* ≤ 2, 0 ≤ *y* ≤ 2) layer oxidized from the mechanically exfoliated 2D layered HfSe_2_ nanosheets acts as the RS medium, sandwiched between the top Ti active electrode and the bottom Au inert electrode. Figure 1b shows the optical images and atomic force microscopy (AFM) image (inset) of the device. The thickness of HfSe*_x_* O*_y_*/HfSe_2_ RS medium is measured to be 18.3 nm.

It is anticipated that the partially oxidized HfSe*_x_* O*_y_* may possess superior performance than the fully oxidized HfO*_x_* because 1) the thin HfSe*_x_* O*_y_* film could remarkably increase the resistance (in contrast to HfSe_2_ which is highly conductive) and thus reduce the operation current of the device; 2) the mixed Se and O, due to their different mobility, could control the shape of the vacancy filament so that its growth and rupture take place only in the “weak” spot which could save the switching energy and increase the cycle‐to‐cycle consistency; and 3) the left‐over HfSe_2_ layer could reduce the Schottky barrier and contact resistance between HfSe*_x_* O*_y_* and the bottom electrode, barring the interdiffusion of ions and vacancies.^[^ ^29^ ^]^ This insulating HfSe*_x_* O*_y_* layer between the top electrode and the 2D HfSe_2_ was acquired by O_2_‐plasma treatment. To select the appropriate O_2_‐plasma treatment time and power, the X‐ray diffraction (XRD) spectra were used to monitor the degree of oxidation. As the oxide layer is usually amorphous, the intensity of XRD peaks could indicate the left‐over HfSe_2_ that has not been oxidized yet. As shown in Figure 1c, the HfSe_2_ nanosheets on the SiO_2_/Si substrate were treated with O_2_ plasma under two different processing times and two different powers. 2D HfSe_2_ has a trigonal crystal structure with strong XRD peaks appearing at 14.3°, 44.1°, and 60.1°, corresponding to the (001), (003), and (004) planes, and according to the (001) diffraction peak, the space between neighboring Hf layers (lattice parameter *c*) can be calculated as 0.618 nm, agreeing with the reported value in refs. ^[^ ^30^ ^]^ and ^[^ ^31^ ^]^. With the increase of O_2_‐plasma time and power, the intensity of the three diffraction peaks decreases, and the energy‐dispersive spectroscopy (EDS) analysis results (Figure S1, Supporting Information) show that the Se/Hf ratio is also subject to a decrease as more of the 2D HfSe_2_ layers have been oxidized. We intend to fabricate thin HfSe*_x_* O*_y_* layers to control the shape of filaments, and hence the full oxidation should be avoided. We selected 60 W and 5 min of the optimized O_2_‐plasma power and time because the XRD peaks of 2D HfSe_2_ using this treatment indicate a partial oxidation. In addition, after this O_2_‐plasma treatment (60 W/5 min), the oxide surface is quite smooth with an average roughness (*R*_a_) of only 2.52 Å (Figure S2, Supporting Information).

The optical transparency of the exfoliated HfSe_2_ flake increases after O_2_‐plasma treatment, as shown in Figure 1d, indicating the formation of oxide layer with larger bandgap. We performed the Raman spectra measurement on HfSe_2_ before and after 5 min O_2_‐plasma treatment to monitor how the material changes. The A_1g_ peaks of pristine HfSe_2_ before the oxidation (red line in Figure 1e) at 199 cm^−1^ are in good agreement with previously reported results.^[^ ^30^, ^32^ ^]^ After 5 min oxygen plasma treatment, the intensity of HfSe_2_ A_1g_ peak position is significantly reduced, and a new HfO*_x_* Raman peak (≈255 cm^−1^) appears^[^ ^33^ ^]^ (blue line in Figure 1e). Raman spectra of HfSe_2_ oxidation by air exposure and O_2_‐plasma treatment with different thicknesses confirm that the oxidation by O_2_‐plasma treatment is more thorough than by spontaneous air exposure, as shown in Figure S3 (Supporting Information). This has also been reflected by X‐ray spectroscopy (XPS) spectra of Hf 4f for air‐exposed and O_2_‐plasma‐treated HfSe_2_ flakes, as presented in Figure 1f, in which the Hf—O bonding (Hf 4f_5/2_ and 4f_7/2_) peaks of plasma‐treated HfSe_2_ samples are located at 18.85 and 17.20 eV, respectively.^[^ ^30^, ^34^ ^]^ As a comparison, two additional Hf core level peaks are located at 18.00 and 16.35 eV, corresponding to the Hf—Se bonds of air‐exposed HfSe_2_.^[^ ^33^, ^34^ ^]^ Therefore, the top surface of HfSe_2_ can be fully oxidized by using O_2_‐plasma treatment. In contrast, the Se 3d_5/2_ and 3d_7/2_ peaks of Se—Hf bonding located at 53.70 and 54.60 eV still contribute most to the chemical bonding of air‐exposed HfSe_2_ (Figure S4, Supporting Information).^[^ ^33^, ^34^ ^]^ On the other hand, for O_2_‐plasma‐treated HfSe_2_, we observed Se 3d_3/2_ (60.13 eV) and Se 3d_5/2_ (59.03 eV) for Se—O bonding, and Se 3d_3/2_ (56.23 eV) and Se 3d_5/2_ (55.28 eV) for Se—Se bonding.^[^ ^33^, ^34^, ^35^ ^]^ We also find that the thinner the starting 2D HfSe_2_ layer is, the easier it is to be oxidized. 2D HfSe_2_ films below 10 nm could be completely oxidized, and hence should be avoided. The as‐fabricated device shows good stability under ambient condition, e.g., the HfSe*_x_* O*_y_* layer on top of HfSe_2_ could protect it from further oxidation in the air.^[^ ^33^ ^]^

### 2.2 RS Behaviors with Low Operation Current below 100 nA

**Figure 2:**
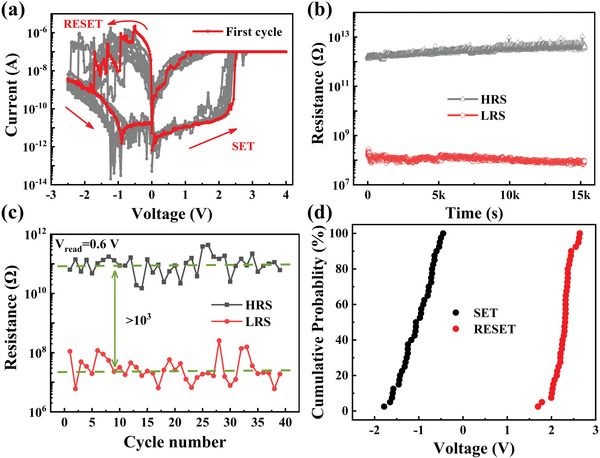
Electrical characterizations of the Ti/HfSe*_x_* O*_y_*/HfSe_2_/Au devices. a) The *I*–*V* curves of the device at low operation current (100 nA). The red curve depicts the first cycle with forming‐free feature, and the subsequent cycles are depicted by the gray curves. b) Good data retention measured at room temperature. Both HRS and LRS were read at 0.1 V. c) Statistical HRS and LRS for 40 DC *I*–*V* sweeps under the 100 nA operation current. The read voltage is 0.6 V. d) Cumulative distributions of the SET/RESET voltages.

Next, the RS behaviors of the fabricated Ti/HfSe*_x_* O*_y_*/HfSe_2_/Au devices were thoroughly characterized. Upon the electrical measurements, the top electrode Ti was biased, while the bottom Au electrode was grounded. As shown in **Figure** **2a**, the device exhibits a repeatable bipolar RS behavior with forming‐free characteristics at an ultralow 100 nA compliance current (*I*_cc_), in which the switching voltage for the first SET operation (red curve in Figure 2a) shows no difference from the subsequent operations. This forming‐free feature is essentially beneficial for large‐scale integration with selective transistors or two‐terminal selectors^[^ ^14^, ^36^, ^37^ ^]^ and the 100 nA operation current is comparable to the lowest operation current in HfO*_x_*‐based memristor reported^[^ ^2^, ^38^ ^]^ as of late. Initially, the device is in a highly insulating state (≈TΩ). As the applied positive voltage increases, the device switches from the high‐resistance state (HRS) to the low‐resistance state (LRS) at ≈2.32 V (SET process). The LRS is nonvolatile as the removal of voltage. A negative bias (≈−2.5 V) can reset the device back to HRS. Moreover, long‐term resistive‐state retention (>15 000 s) of both HRS and LRS and more than 40 DC switching cycles at 100 nA with a large on/off ratio (10^3^) are demonstrated in Figure 2b,c. Similar RS characteristics can be reproduced in other devices (Figure S5, Supporting Information). The SET and RESET voltage statistics for the 40 cycles exhibit a normal distribution centered at 2.32 and −0.7 V, respectively (Figure 2d), showing fair cycle‐to‐cycle consistency. Moreover, good thermal stability can broaden the potential applications of memristors in harsh environment.^[^ ^11^, ^16^ ^]^ As shown in Figure S6 (Supporting Information), *I*–*V* curves at elevated temperatures (from room temperature to 400 K) and the retention of both HRS and LRS at 85 °C show good thermal stability of the devices which remain functional even at high temperature.

**Figure 3:**
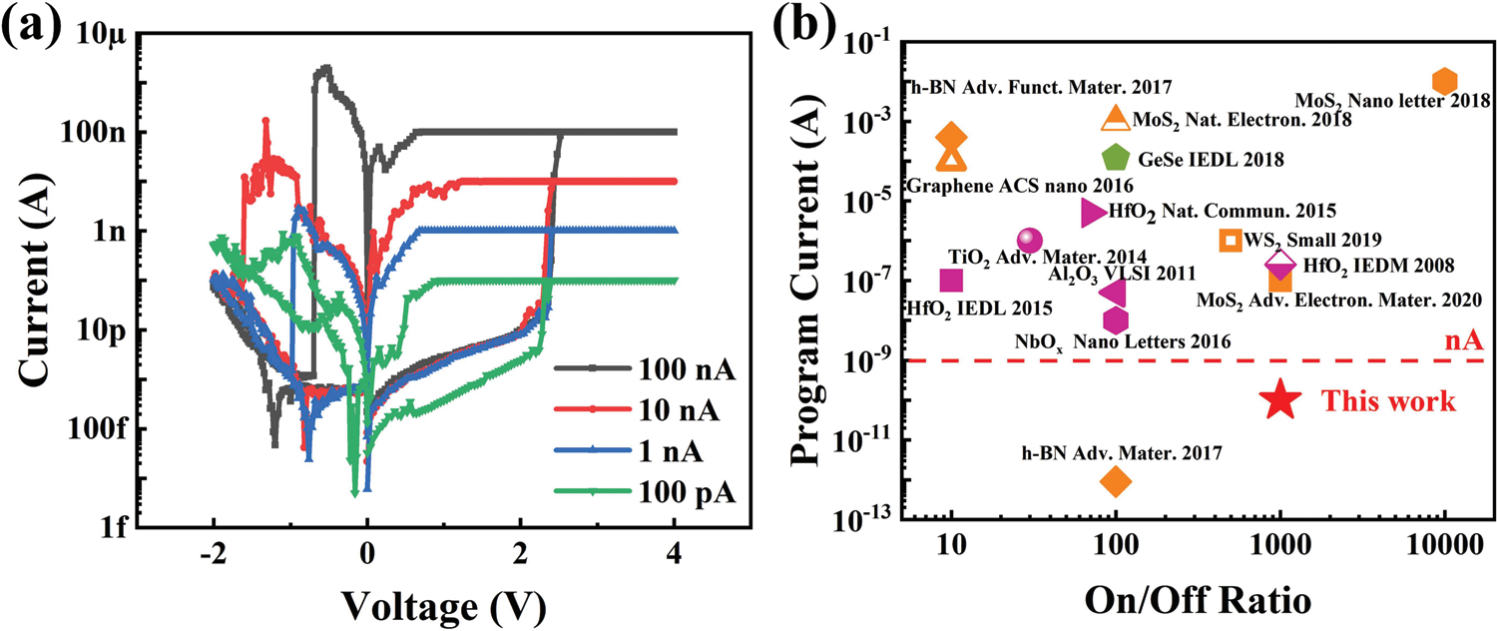
a) Nonvolatile RS with different *I*_cc_ of 100 nA, 10 nA, 1 nA, and 100 pA. b) Comparison of the operation current and on/off ratio of our device (red star) with other chalcogenide‐based memristors (green symbol), transition metal oxides‐based memristors (purple symbols), and 2D materials‐based memristors (orange symbols).

To push the limit of operation current of our devices, *I*–*V* curves with even lower *I*_cc_ were carefully measured, as shown in **Figure** **3a**. The device remains functional with an on/off ratio of 260× when the SET current is reduced to a striking low sub‐nA (100 pA) range, which is much lower than reported values in traditional chalcogenide memristors,^[^ ^39^ ^]^ transition metal oxides based memristors^[^ ^10^, ^37^, ^38^, ^40^, ^42^ ^]^ and most 2D materials based memristors^[^ ^16^, ^20^, ^21^, ^43^, ^44^, ^45^, ^46^ ^]^ (Figure 3b). Even in such a low‐current mode, the device still shows good data retention without large drift over time (Figure S7, Supporting Information).To estimate the energy required for each operation, we applied voltage pulses to set and reset the devices (Figure S8, Supporting Information). Our devices achieve the SET and RESET speed of less than 10 µs and 50 ns, and the energy required for SET (4 V/10 µs) and RESET (−4 V/50 ns) operations are estimated to be 160 aJ and 114 fJ, respectively, approaching the energy consumption of the state‐of‐the‐art memristors.^[^ ^19^, ^47^ ^]^ Note that the size of our device is 5 × 5 µm^2^, and thus further reduction of programming energy is anticipated by downscaling the device.^[^ ^13^, ^48^ ^]^

### 2.3 Mechanisms of Low‐Power RS Behavior

**Figure 4:**
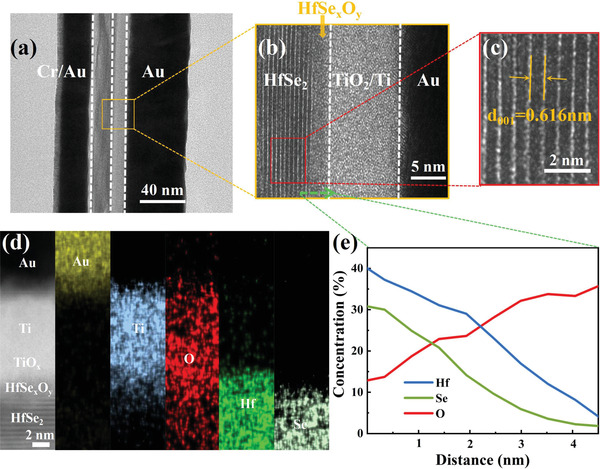
a) Cross‐sectional TEM image of the device. b) Amplified HAADF image of the device structure in the selected TEM imaging of (a). c) Amplified TEM image of layered HfSe_2_ in (b), showing that the interlayer distance along [001] axis of the trigonal HfSe_2_ is 0.616 nm. d) Cross‐sectional TEM image of the Ti/HfSe*_x_* O*_y_*/HfSe_2_/Au heterostructure and EDS elemental mapping of Au, Ti, Hf, Se, and O. e) EDS line scan across the HfSe*_x_* O*_y_* layer, along the green arrow in (b).

To investigate the RS mechanism in the HfSe*_x_* O*_y_* layer, the transmission electron microscopy (TEM) and EDS analyses were used to unravel the structural and compositional variation of the device. **Figure** **4a**–c clearly shows that amorphous HfSe*_x_* O*_y_* layer with a thickness of ≈2 nm is formed, with a clear interface connecting to the layered 2D HfSe_2_ which maintains the trigonal crystal structure with an interlayer distance of 6.16 Å along [001] axis, consistent with previous XRD results (6.18 Å). EDS line profile in Figure 4e shows that when approaching Ti electrode, the content of Se decreases and the content of O increases. A thin Ti‐oxide layer is intentionally formed between Ti/HfSe*_x_* O*_y_* by drawing a few O atoms from HfSe*_x_* O*_y_*, leaving empty O vacancies to form conductive filaments.^[^ ^10^, ^49^ ^]^ In a contrast experiment by replacing Ti with Au, the fabricated Au/HfSe*_x_* O*_y_*/HfSe_2_/Au device shows unipolar switching behavior which bears large variation and low endurance (Figure S9, Supporting Information), indicating insufficient O vacancies without Ti electrode. It is known that Se is less mobile than O, and thus the diffusion of vacancies becomes more sluggish near the HfSe_2_ side (left side in Figure 4e) where Se is richer than O, leading to thinner vacancy filament than that forms near the top layer.

**Figure 5:**
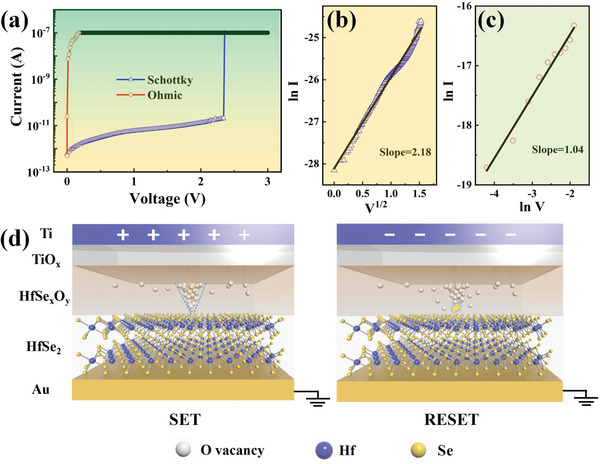
Mechanisms of RS behavior. a–c) The Schottky emission and Ohmic fitting for the HRS and LRS in the positive part of the *I*–*V* curve. d) The cone‐shaped O‐vacancy filament is formed and ruptured at the tip on SET and RESET processes in our device, requiring ultralow energy.

The *I*–*V* curves fitted in **Figure** **5a**–c show that the HRS is governed by the Schottky emission conduction mechanism with a slope of 2.18, while the LRS exhibits Ohmic conduction behavior with a slope of 1.04. The Schottky emission model is usually used to describe the conduction mechanism of dielectric films,^[^ ^50^, ^51^ ^]^ agreeing with the TiO*_x_* and insulating HfSe*_x_* O*_y_* layer at HRS. The active Ti electrode grabs some O^2−^ from HfSe*_x_* O*_y_* layer to form TiO*_x_*, enriching the O vacancies near the interface. As the device is positively biased (left panel in Figure 5d), the positively charged O vacancies move toward the bottom electrode, and are eventually barred by the 2D HfSe_2_, forming an O‐vacancy conductive channel across the HfSe*_x_* O*_y_* layer. Owing to the gradient concentration of O and Se across the HfSe*_x_* O*_y_* layer (Figure 4e), the number of O vacancies near the Ti electrode is much higher than that near HfSe_2_ layers, and thus the conductive channel is likely to be “cone” shaped.^[^ ^52^ ^]^ The HfSe_2_ layer, with larger vacancy formation energy than amorphous HfSe*_x_* O*_y_*, acts as a “wall” to prevent the accumulation of O vacancies at the cathode, so that this conic filament maintains a good shape. The conductive channel behaves as an Ohmic resistor, complying with the fitting result in Figure 5c. Oppositely, when we apply negative voltage to reset the device (right panel in Figure 5d), the conductive filament is ruptured by retrieving the O vacancies from the tip of the cone, and this process requires very low energy.

### 2.4 Implementation of Low‐Power Boolean Logic for In‐Memory Computing

**Figure 6:**
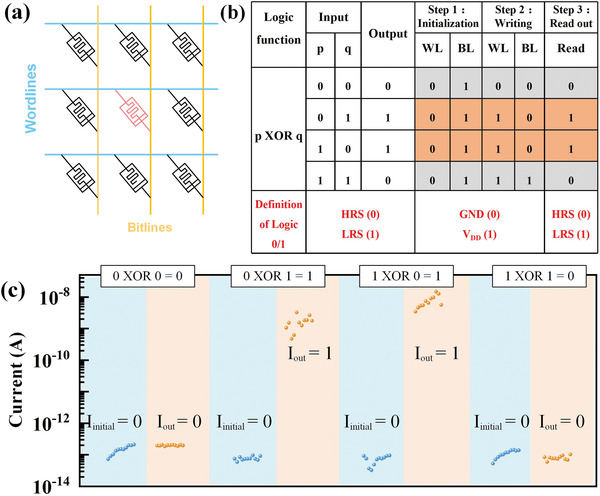
a) Schematic of the logic circuits. WL is connected to the top electrode and BL is connected to the bottom electrode. b) XOR logic and operation steps are shown in the truth table. c) Experimental results of XOR logic operations with the reading voltage of 0.1 V.

In‐memory computing emerges as a non‐von Neumann computation paradigm that can gain significant improvements in computation efficiency, especially for data‐intenstive tasks.^[^ ^53^ ^]^ Memristive bitwise logic computation is an important class of in‐memory computing method, generally on par with memristive analog computation.^[^ ^6^, ^54^ ^]^ Here, by adopting the four‐variable based sequential logic method,^[^ ^55^, ^56^ ^]^ we demonstrate that the HfSe_2_‐based memristor could be a good candidate for low‐power Boolean logic computation. Any single device in the crossbar array (**Figure** **6a**) can be selected to perform the required logic function in three steps: initialization, writing operation and readout. In the initialization step, the physical variable *W* is executed to determine the initial state of the device. HRS represents logical “0,” and LRS represents logical “1.” A writing operation step follows the initialization step by input of the rest three physical variables (*A*, *B*, and *C*). In particular, two physical variables A and B are operation signals to determine the voltage potential of WL and BL. Zero potential (grounded) represents logical “0,” and high potential (*V*_dd_) represents logical “1.” The last physical variable *C* is represented by the applied path of *A* and *B*. *C* = 1 is defined as *A* is applied to WL and *B* to BL, while *C* = 0 is defined as *A* is applied to BL and *B* to WL. Through above two steps, the logic results can be read out by a nondestructive read step with a bias of *V*_read_. Hence, the logic output is given by the following equation

(1) $$L\;=\;W\cdot \left(\bar{\bar{A}\cdot B\cdot C+A\cdot \bar{B}\cdot \bar{C}}\right)+\bar{W}\cdot \left(A\cdot \bar{B}\cdot C+\bar{A}\cdot B\cdot \bar{C}\right)$$

Taking the XOR function of two input variables *p* and *q*, a functionally complete logic in Boolean system, for instance, four variables are assigned (*W* = 0, *A* = *p*, *B* = *q*, and *C* = *p*), respectively. We experimentally demonstrate its implementation through pulse modulation at the pulse voltages of 4 V and pulse width of 10 µs. In detail, the logic could be executed by the following steps: 1) In the initialization step (*W* = 0), regardless of the input combination (*p* and *q*), the selected device is always initialized to HRS by applying 0 V and *V*_dd_ to corresponding WL and BL, respectively. 2) In the writing operation step (*A* = *p*, *B* = *q*, and *C* = *p*), when *C* = *p* = 0, *A* (*p*) and *B* (*q*) are assigned to BL and WL, respectively; When *C* = *p* = 1, the direction of *A* and *B* is reversed. 3) The logic results, represented by the final resistance state of the memristor, can be read out by a small read voltage (100 mV in this work) and transmit to the next stage computation. Figure 6c shows the experimental results of four possible input combinations for the XOR logic, which fit well with the truth table. Our devices are functional at very low operation currents, compared with previous studies.^[^ ^57^, ^58^, ^59^ ^]^ Figure S10 (Supporting Information) further demonstrates the operation methods and experimental results for other two important Boolean logic functions: IMP and NAND, which provides more evidences on the application potential of energy‐efficient in‐memory computing.

## 3 Conclusion

In summary, we fabricate an energy‐efficient memristive logic device based on HfSe_2_ oxides. Ultrathin HfSe*_x_* O*_y_* layer on the surface of mechanically exfoliated 2D HfSe_2_ nanosheets is demonstrated to be an excellent RS medium. The fabricated Ti/HfSe*_x_* O*_y_*/HfSe_2_/Au devices exhibit repeatable bipolar RS behavior with forming‐free characteristics. The high‐quality HfSe*_x_* O*_y_* layer acquired by O_2_‐plasma treatment significantly increases the resistance of the devices, thus leading to a low operation current down to 100 pA. The resulting power consumption reaches 0.1 fJ to 0.1 pJ, much lower than most reported memristors. Such a low programming energy stems from the efficient switching mechanism by forming and rupture of cone‐shaped O‐vacancy filaments, as induced by the gradient concentration of O and Se in the HfSe*_x_* O*_y_* layer. Low‐power Boolean logic functions using our device are realized toward future applications in the in‐memory computing.

## 4 Experimental Section

### Device Fabrication

The device was fabricated by first patterning the bottom electrode (25 nm Au and 5 nm Cr) on Si substrates with 300 nm thick SiO_2_ layers. Layered HfSe_2_ was then exfoliated and transferred on the bottom electrode. For the dry‐oxidation treatment, the flakes were introduced into a vacuum chamber (PDC‐MG) with O_2_ plasma at a power of 60 W for 5 min, forming the ≈2 nm thick HfSe*_x_* O*_y_* film from the topmost HfSe_2_ layers. Finally, the top electrode (10 nm Ti and 40 nm Au) was then deposited on top of the HfSe*_x_* O*_y_*/HfSe_2_ heterostructure. The crosspoint area of the memristor is 5 × 5 µm^2^. The top and bottom electrodes were patterned by lithography (MA8/BA8 Gen4) and deposited by e‐beam evaporator (Ohmiker‐50B).

### Materials Characterizations

Optical microscope (MOTIC BA310Met) and AFM (Bruker Dimension Edge) under tapping mode were used to characterize the surface morphology, size, and thickness of the 2D materials. Raman spectra were acquired using a confocal Raman microscope (Horiba Jobin‐Yvon LabRAM HR800) with an excitation wavelength of 532 nm, and a laser with a moderate power of 0.5 mW was selected. XPS were carried out using an AXIS‐ULTRA DLD‐600W instrument. XRD measurements were performed on a Bruker D2 PHASER Diffractometer with Cu‐K*α* radiation (*λ* = 0.154 nm). The high‐resolution TEM (HRTEM) and EDS mapping were carried out at an acceleration voltage of 200 kV (FEI Titan Themis 200 TEM with a Bruker Super‐X EDX system). TEM samples were prepared using EI Helios 450s dual beam FIB system.

### Electrical Measurements

The electrical characterization includes *I*–*V* curves, retention and endurance properties measured using a Cascade probe station. The DC measurements and voltage pulse operation in the AC measurements were performed using an Agilent B1500A semiconductor analyzer. During the measurements, the bias voltage was applied to the Ti top electrode and the bottom electrode Au was grounded.

## Conflict of Interest

The authors declare no conflict of interest.

## Supporting information

Supporting Information

## Data Availability

All data are available in the manuscript or the Supporting Information, and are available from the corresponding authors upon reasonable requests.
