# Supplementary material for: Low‐Power Memristive Logic Device Enabled by Controllable Oxidation of 2D HfSe2 for In‐Memory Computing
Source: Adv Sci (Weinh). 2021 May 29;8(15):2005038. doi: 10.1002/advs.202005038 (PMC8336485; doi:10.1002/advs.202005038)
Supplement: Supplementary file 1 — Supporting Information [file ADVS-8-2005038-s001.pdf]

© 2021 Wiley-VCH GmbH

Supporting Information

for *Adv. Sci.,* DOI: 10.1002/advs.202005038

Low-power Memristive Logic Device Enabled by Controllable Oxidation of Two-dimensional HfSe2 for in-memory Computing

Long Liu,[#] *Yi Li,*[#] *Xiaodi Huang, Jia Chen, Zhe Yang, Kan-Hao Xue, Ming Xu,** *Huawei Chen, Peng Zhou,** and *Xiangshui Miao**

Supporting Information

Low-power memristive logic device enabled by controllable oxidation of two-dimensional $HfSe_2$ for in-memory computing

Long Liu,[#] *Yi Li,*[#] *Xiaodi Huang, Jia Chen, Zhe Yang, Kan-Hao Xue, Ming Xu,** *Huawei Chen, Peng Zhou,** and *Xiangshui Miao**

Long Liu, Prof. Yi Li, Xiaodi Huang, Jia Chen, Dr. Zhe Yang, Prof. Kan-Hao Xue, Prof. Ming Xu, Prof. Xiangshui Miao
Wuhan National Laboratory for Optoelectronics, School of Optical and Electronic Information, Huazhong University of Science and Technology, Wuhan 430074, China
Email: mxu@hust.edu.cn, miaoxs@hust.edu.cn

Huawei Chen, Prof. Peng Zhou
State Key Laboratory of ASIC and System, School of Microelectronics, Fudan University, Shanghai 200433, China
Email: pengzhou@fudan.edu.cn,

[#]Authors contribute equally to this work
[*]E-mail: mxu@hust.edu.cn, pengzhou@fudan.edu.cn, miaoxs@hust.edu.cn

Keywords: 2D materials, $HfSe_2$, oxidation, memristor, low power consumption, nonvolatile resistive switching, in-memory computing

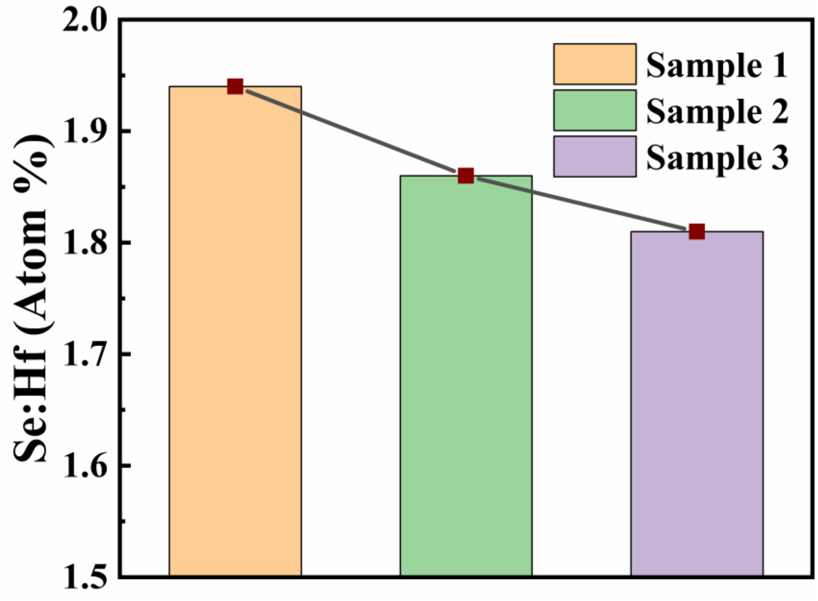

Figure S1. With the increase of oxidation power, the Se:Hf ratio gradually decreases. Sample 1 (air exposure), Sample 2 (60 W, 5 min), Sample 3 (140 W, 5 min)

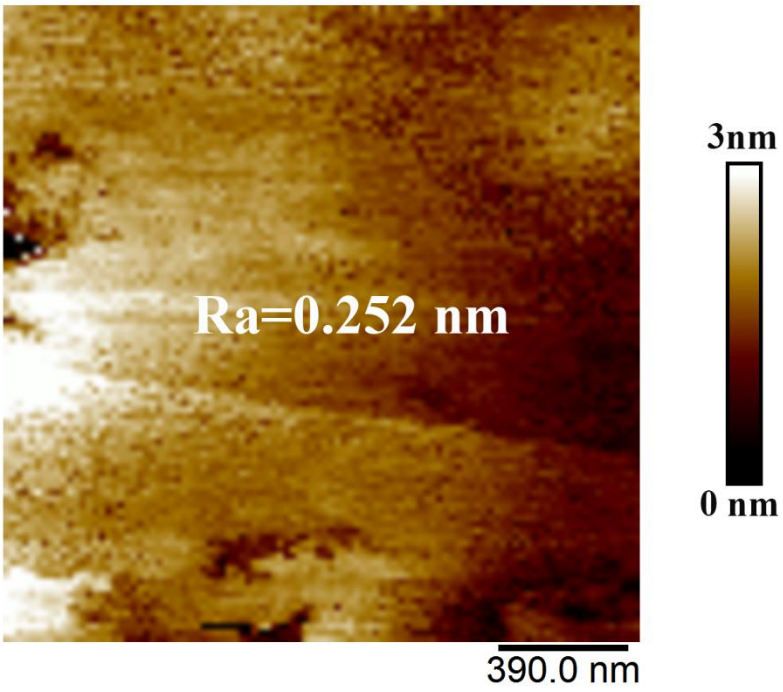

Figure S2. After O_2-plasma treatment (60 W, 5 min), the oxide surface remains smooth, with an average roughness (Ra) of only 2.52 Å, measured by atomic force microscopy. The scanning area is 3×3 μm^2.

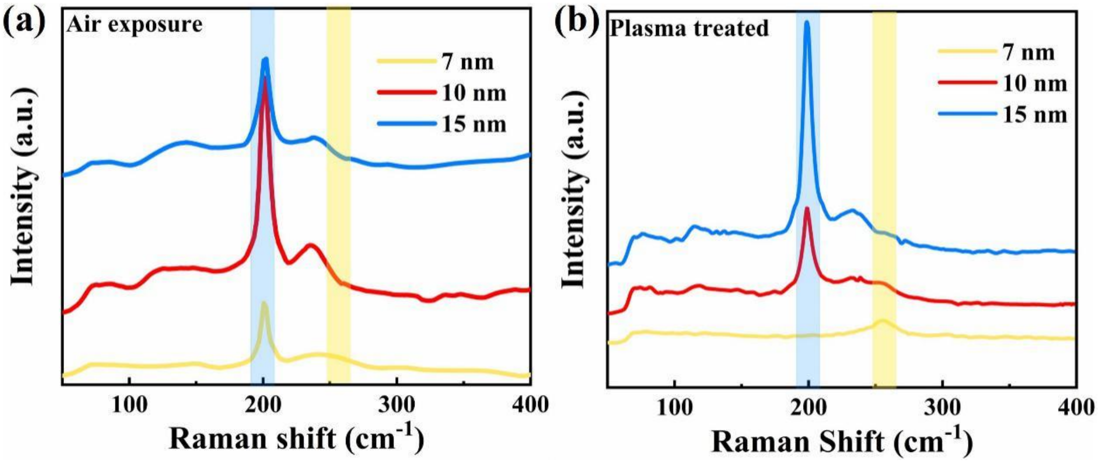

Figure S3. Raman spectra of air-exposed and O_2-plasma treated of $HfSe_2$ with different thicknesses. Oxidation by O_2-plasma treatment is more thorough than by spontaneous air exposure. The thinner of the starting 2D $HfSe_2$ layer, the easier it is to be oxidized, and 2D $HfSe_2$ films below 10 nm have been completely oxidized.

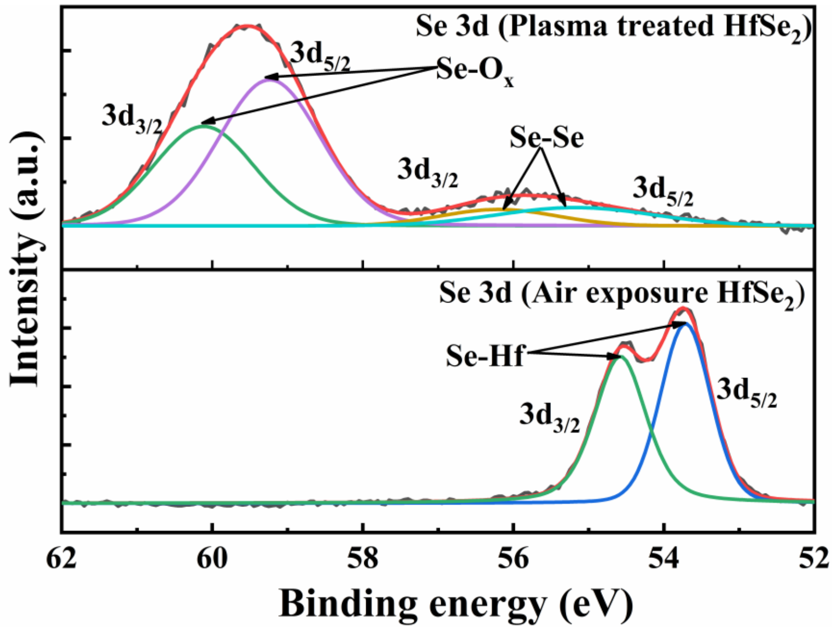

Figure S4. XPS spectra of Se 3d core level for air exposure and oxygen-plasma treated $HfSe_2$ flakes. The two different chemical states of O_2 plasma treated $HfSe_2$ indicate the formation of the residual materials including SeO_x and Se.

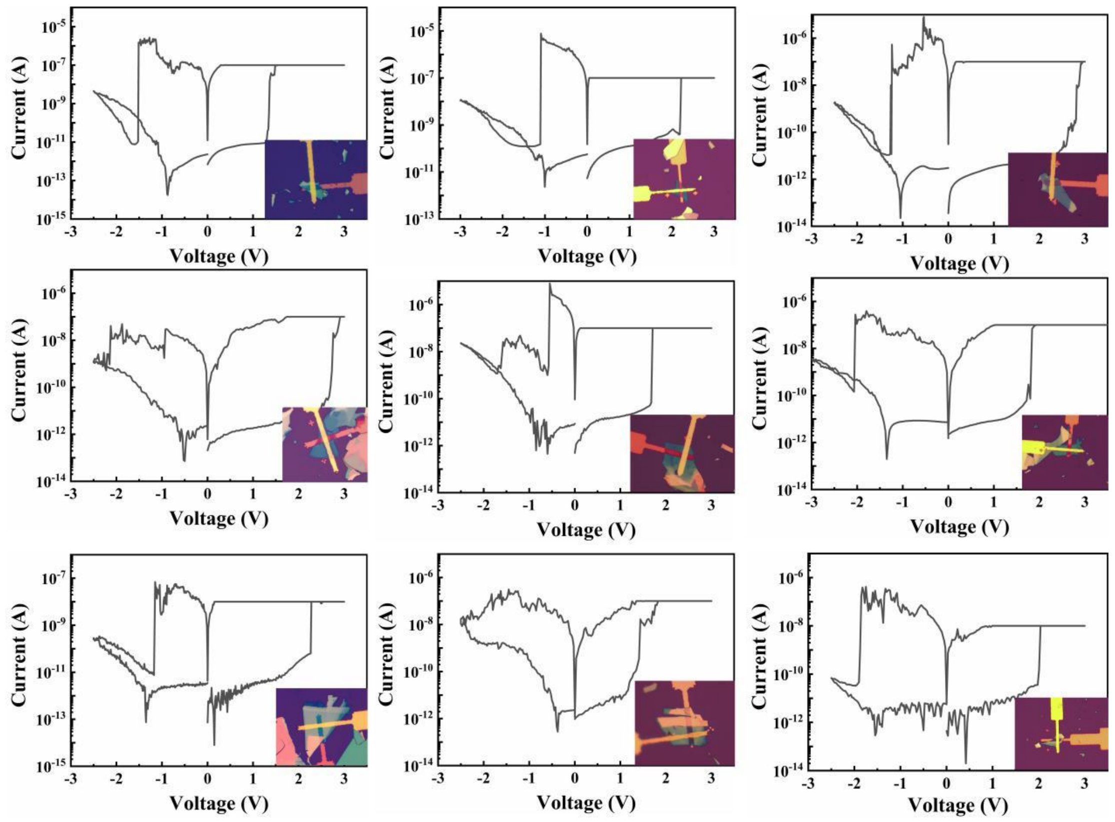

Figure S5. I-V curves of multiple devices. All devices can switch at 100 nA operation current or less within 3V.

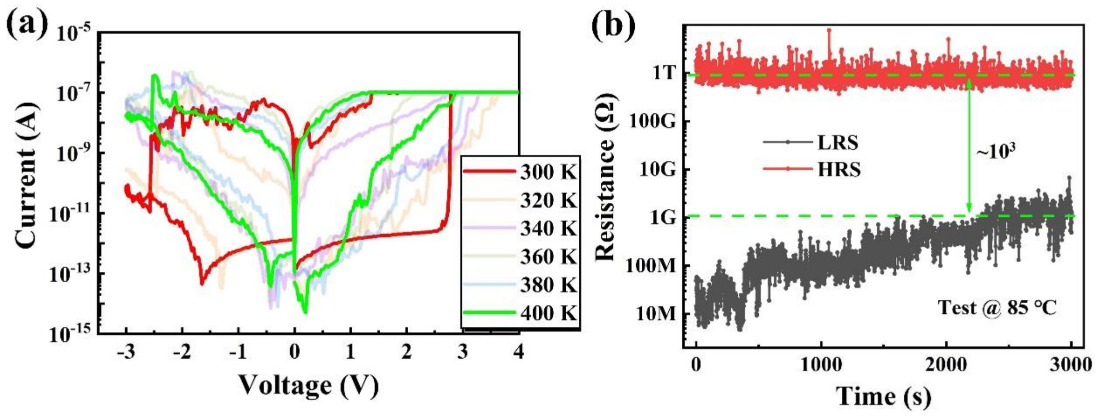

Figure S6. Thermal stability of the devices**.** a) I-V curves of a typical device at elevated temperature up to 400 K, showing functional switching behavior. b) The data retention of both HRS and LRS at 85 °C.

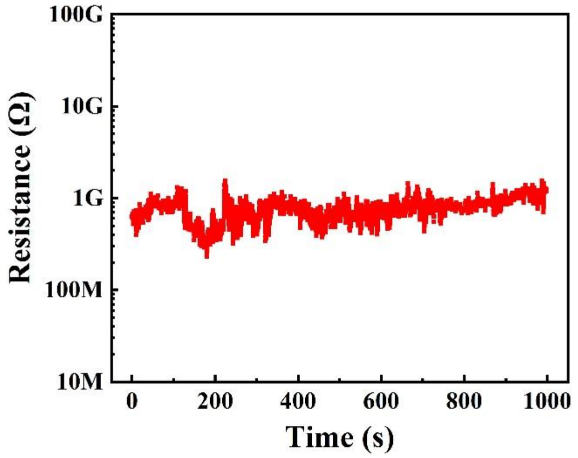

Figure S7. Retention characteristics of the LRS under 100 pA operation current at room temperature using the read voltage of 10 mV.

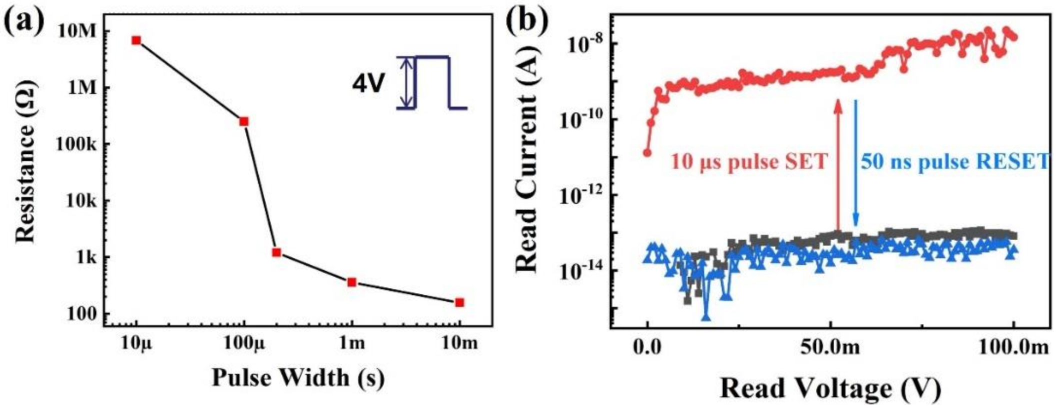

Figure S8. a) The LRS decreases with the increase in the SET pulse width, when the SET pulse amplitude is fixed at 4 V. The SET speed of the device can be less than 10 μs. b) Read operation after applying 4 V/10 μs SET and -4 V/50 ns RESET operation pulses. Both HRS and LRS are read at 0.1 V.

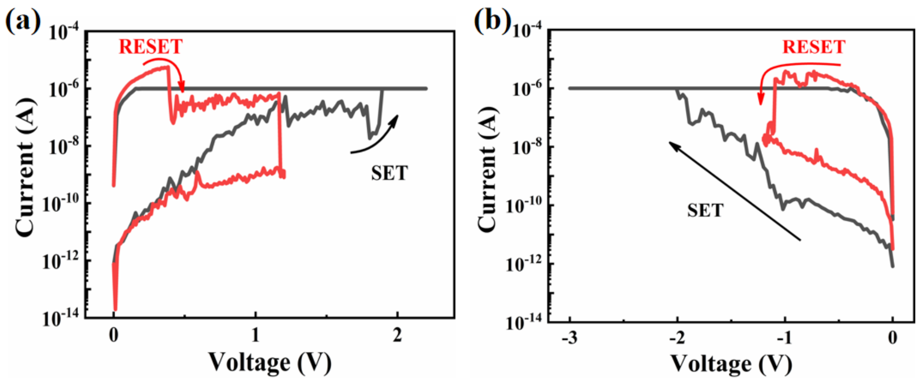

Figure S9. Unipolar RS behavior of the Au/$HfSe_xO_y$/$HfSe_2$/Au device in both positive and negative sweeps. This device with symmetric metal electrodes shows unreliable switching behaviors with large variation and limited endurance.

(a)

Logic operation	Input		Output	Step 1 : Initialization		Step 2 : Writing		Step 3 : Read out
	p	q		WL	BL	WL	BL	Read
p IMP q	0	0	1	1	0	0	0	1
	0	1	0	1	0	1	0	1
	1	0	1	1	0	0	1	0
	1	1	1	1	0	1	1	1
p NAND q	0	0	1	1	0	0	0	1
	0	1	1	1	0	0	0	1
	1	0	1	1	0	1	0	1
	1	1	0	1	0	0	1	0

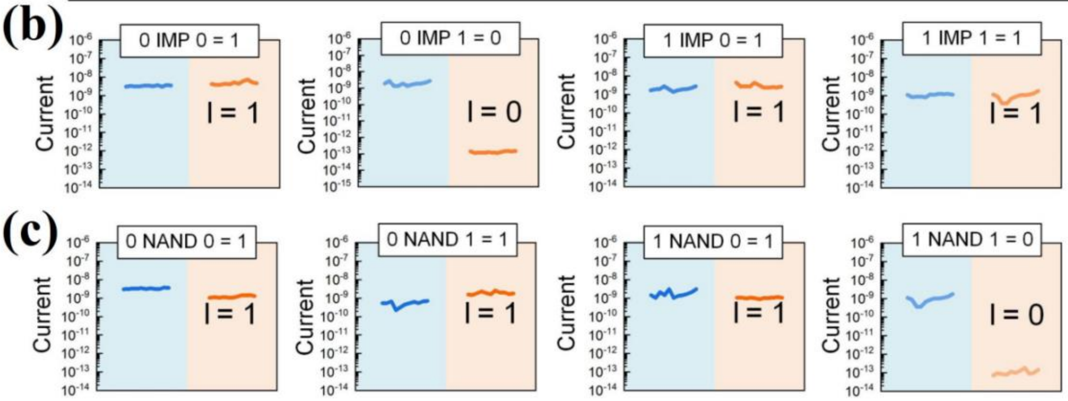

Figure S10. a) IMP and NAND logic functions and corresponding operation steps are shown in the truth table. For IMP and NAND logic functions, four variables are assigned (W=1, A=p, B=q and C=0) and (W=1, A=0, B=p and C=q) respectively. c) Experimental results of IMP and NAND logic operations, demonstrating the feasibility of our logic method and the functional device.
